# Nontargeted Screening Using Gas Chromatography–Atmospheric Pressure Ionization Mass Spectrometry: Recent Trends and Emerging Potential

**DOI:** 10.3390/molecules26226911

**Published:** 2021-11-16

**Authors:** Xiaolei Li, Frank L. Dorman, Paul A. Helm, Sonya Kleywegt, André Simpson, Myrna J. Simpson, Karl J. Jobst

**Affiliations:** 1 Department of Chemistry, Memorial University of Newfoundland and Labrador, St. John’s, NL A1C 5S7, Canada; xiaolei.li@mun.ca; 2 Department of Chemistry, The Pennsylvania State University, University Park, State College, PA 16802, USA; fld3@psu.com; 3 Department of Chemistry, Dartmouth College, Hannover, NH 03755, USA; 4 Ontario Ministry of Environment, Conservation and Parks, Toronto, ON M4V 1M2, Canada; Paul.Helm@Ontario.ca (P.A.H.); Sonya.Kleywegt@ontario.ca (S.K.); 5 Departments of Chemistry and Physical & Environmental Sciences, University of Toronto, Toronto, ON M1C 1A4, Canada; andre.simpson@utoronto.ca (A.S.); myrna.simpson@utoronto.ca (M.J.S.)

**Keywords:** GC–API, GC–APCI, GC–APLI, GC–APPI, GC–MS, persistent organic pollutants, nontargeted screening, computational mass spectrometry

## Abstract

Gas chromatography–high-resolution mass spectrometry (GC–HRMS) is a powerful nontargeted screening technique that promises to accelerate the identification of environmental pollutants. Currently, most GC–HRMS instruments are equipped with electron ionization (EI), but atmospheric pressure ionization (API) ion sources have attracted renewed interest because: (i) collisional cooling at atmospheric pressure minimizes fragmentation, resulting in an increased yield of molecular ions for elemental composition determination and improved detection limits; (ii) a wide range of sophisticated tandem (ion mobility) mass spectrometers can be easily adapted for operation with GC–API; and (iii) the conditions of an atmospheric pressure ion source can promote structure diagnostic ion–molecule reactions that are otherwise difficult to perform using conventional GC–MS instrumentation. This literature review addresses the merits of GC–API for nontargeted screening while summarizing recent applications using various GC–API techniques. One perceived drawback of GC–API is the paucity of spectral libraries that can be used to guide structure elucidation. Herein, novel data acquisition, deconvolution and spectral prediction tools will be reviewed. With continued development, it is anticipated that API may eventually supplant EI as the de facto GC–MS ion source used to identify unknowns.

## 1. Introduction

Chemistry is essential to the modern world, producing molecules and materials required in all facets of society. Tens of thousands of chemical substances, representing millions of individual chemical compounds, have now been introduced to the global market and this number is increasing [1]. Since 1982, the number of substances registered in the US Toxic Substances Control Act Chemical Substance Inventory (TSCA) has grown from 62,000 to over 86,557 [2]. According to the United Nations Global Chemicals Outlook [3], the total volume of chemicals is expected to increase at a rate that outpaces population growth over the next decade. Concerns that some of these chemicals can persist in the environment, bioaccumulate and adversely impact human health have led to international efforts to restrict the (un)intentional release of 28 groups of hazardous chemicals, i.e., persistent organic pollutants (POPs) [4]. However, the number of unregulated POPs may be much larger. A recent evaluation of substances compiled in the TSCA and other national chemical inventories has resulted in a list of 3421 chemical substances that may be persistent and bioaccumulative, and have so far evaded detection in the environment. The identification of unknown pollutants, most appropriately using mass spectrometry, is the critical first step to evaluating their potential harm to the environment, establishing policies and guidelines to limit exposure, and preventing global contamination. Such experiments have been coined nontargeted screening (NTS) [5]. Compared to target screening and suspect screening that involve completely or partly known prior information of the compounds in the sample, nontargeted screening is performed without knowledge of exact mass, isotope, adduct or fragmentation behaviour [6].

Gas chromatography– and liquid chromatography–high-resolution mass spectrometry (GC–HRMS and LC–HRMS) are powerful, complementary techniques for NTS of persistent and bioaccumulative organic pollutants. The need for both techniques is underlined by the results of a recent interlaboratory study led by Rostkowski et al. [7,8]. The analysis of an indoor dust sample by over 20 different laboratories using both GC–HRMS and LC–HRMS resulted in the tentative identification of 2350 compounds. Of these compounds, approximately half were identified by GC–HRMS and only 5% of the compounds were detected using both GC and LC. This is because the compounds amenable to GC or LC separation often have different volatility, polarity and ionization behavior. While LC–HRMS has attracted more recent attention, comprehensive NTS of environmental samples cannot be performed without GC–MS.

Most GC–MS instruments employ electron ionization (EI), which produces highly reproducible, structure-diagnostic mass spectra of organic pollutants. A pollutant’s identity can be established by searching databases of experimental EI spectra (70 eV) such as the NIST Mass Spectral Library [9]. The use of lower ionization energies in EI typically results in a significant decrease in sensitivity and for this reason is rarely used. However, extensive fragmentation under EI conditions can also produce mass spectra in which the molecular ion is absent, thereby confounding elemental composition determination and structure elucidation. This drawback can potentially be solved by lowering the electron energy in EI or using other vacuum ionization techniques such as chemical ionization (CI) [10], photoionization (PI) [11] and field ionization (FI) [12], which impart significantly less energy to the analyte molecules. The past 30 years have also witnessed the advent of atmospheric pressure ionization techniques and (hybrid) mass analyzers, whose development was primarily driven by the need for LC–MS and direct analysis applications. An unintended consequence is that the concept of performing “GC–MS on an LC–MS instrument” [13] has attracted renewed interest. GC and comprehensive two-dimensional gas chromatography (GC×GC) have recently been hyphenated with a variety of atmospheric pressure ionization sources, including: atmospheric pressure chemical ionization (APCI) [14]; atmospheric pressure photoionization (APPI) [15]; atmospheric pressure laser ionization (APLI) [16]; and electrospray ionization (ESI) [17]. Ionization at elevated pressures offers several advantages. Collisional cooling can often minimize fragmentation and increase the yield of (quasi)molecular ions [18]. The resulting detection limits can be approximately 10~100 times lower than conventional GC–MS experiments [19]. Modifying an LC–MS instrument to perform GC–MS may also reduce costs by minimizing the number of specialized instruments required for analysis. The most attractive benefit, however, may be the fact that GC–API can be adapted to mass analyzers [20] and ion mobility mass spectrometers [21] that result in novel configurations that could potentially better tackle complex (environmental) mixtures. GC–APCI has been successfully applied to complex mixtures, such as phenolic compounds in olive oil [22], metabolites of avocados [23], fatty acids in fish [24], exhaled volatile organic compounds [25], and steroid hormone profiles in human breast adipose tissue [26].

In this review, we summarize the recent developments and applications of GC–API reported during the last five years, using SciFinder with the keywords “GC–APCI”, “APGC”, “GC–APPI”, “GC–APLI” or “GC–ESI”. The ion source designs, geometries and ionization mechanisms have been reviewed elsewhere [19,27]. Our contribution will instead focus on the application of various GC–API techniques to identify unknown pollutants, as well as the computational techniques being developed to predict mass spectra and aid in the interpretation of NTS data.

## 2. Gas Chromatography–Atmospheric Pressure Ionization Techniques

Gas chromatography–atmospheric pressure chemical ionization (GC–APCI) was first developed in the 1970s [14]. It saw only limited usage until McEwan & Mckay [28] and Schiewek et al. [29] adapted the approach to commercially available LC–MS instruments in the early 2000s. The ensuing years witnessed the development of various GC–API ion sources adapted from LC–MS applications. Atmospheric pressure photoionization (APPI), first developed by Robb et al. [30] and Syage et al. [31] as an LC–MS ion source, was later adapted by Revelsky et al. [15] for GC–MS. Schiewek et al. [16] developed an atmospheric pressure laser ionization (APLI) source for GC–MS in 2007 and Brenner et al. [17] were the first to employ an electrospray ionization (ESI) emitter to promote ionization of GC effluent. In 2005 [32], Cody introduced Direct Analysis in Real-Time (DART), a technique that makes use of Penning ionization, wherein a molecule is ionized through collision with an electronically excited (metastable) atom. In 2008, the DART ion source was first hyphenated with GC [33], although a similar technique called metastable atom bombardment (MAB) had been developed as a vacuum ion source almost a decade earlier [34]. Dielectric barrier discharge (DBD) ionization is a recent innovation in GC–MS ion source development [35]. DBD ionization occurs in a low-temperature plasma and this approach has been developed in parallel for LC, GC and direct analysis modes of operation. The ion source designs, geometries, and mechanisms of various GC–API techniques have been reviewed elsewhere [11,18,27]. In the following sections, the techniques are briefly summarized, and their advantages and limitations are discussed in light of the results of recent publications in Table 1.

### 2.1. Atmospheric Pressure Chemical Ionization (APCI)

Ionization under APCI conditions is a seemingly straightforward process. When GC effluent exits the column, a high flow of make-up gas from the transfer line sweeps the GC effluent towards the corona discharge. A plasma consisting of primary ions (e.g., N_2_^•+^ and N_4_^•+^ when using N_2_ as the make-up gas) and electrons is generated. Analyte molecules (M) may undergo charge exchange with the primary ions if the recombination energy of the primary ions exceeds the ionization energy (IE) of M. In the same vein, the formation of radical anions (M^•−^) will also occur if the electron affinity (EA) of M is sufficiently high. Internal energy in the incipient ions is quickly dissipated by non-reactive collisions with the surrounding make-up gas, thus minimizing fragmentation. This process also depends on the nature of the reagent gas and the presence of other compounds or ions that may be added as dopants.

Nitrogen is the most common reagent gas used in GC–APCI. This is partly because it is conveniently and inexpensively produced (usually by purification of compressed air) at a rate that is necessary for operation of most API ion sources. Although nebulization and desolvation of liquid droplets is not a concern in GC–API, a relatively high volume of nitrogen is still consumed as a skimmer or curtain gas around the orifice of the mass spectrometer to impede neutrals from entering. The ionization energy of N_2_ (IE = 15.6 eV) also exceeds that of most organic molecules, making N_2_ ideal for the analysis of a wide range of compounds, as is often required for NTS. In some cases, it may be desirable to select a gas with an ionization energy that lies above that of the analyte, and below that of potential interferents. However, in practice, the high consumption of gases by most API sources discourages the use of alternative gases that are more expensive than nitrogen.

Efforts to control the introduction of reagent gases and dopants have mostly been restricted to placing a small vial containing a volatile liquid into the ion source, sometimes with a short piece of capillary tube inserted in the vial’s septum to restrict the flow. For example, when H_2_O or other protic solvents (S) are introduced to the ion source, they may undergo charge exchange to form (H_2_O^•+^ or S^•+^), and subsequently self-protonate to form H_3_O^+^ or SH^+^ according to the reaction: S^•+^ + S → [S − H]^•^ + SH^+^. The protonated solvent molecule SH^+^ may then transfer its proton to an analyte molecule to form [M + H]^+^ if the proton affinity of M exceeds that of S. The formation of [M + H]^+^ ions by protonation instead of charge exchange may be desirable in cases where the compounds of interest have relatively high proton affinities compared to their potential interferents. Schreckenbach et al. used this approach to confirm the identity of the molecular ion of a previously unknown chlorinated amide [20]. However, the unintentional or uncontrolled introduction of H_2_O (e.g., from laboratory air humidity) can be a nuisance, especially if the compound of interest has a low PA and thus can be suppressed under “wet” conditions.

In the negative mode, M can form M^•−^ radical anions through electron capture negative ionization. The presence of low concentrations of oxygen (<1%) may also result in displacement reactions between M and O_2_^•−^ to form ions [M − X + O]^•−^ (where X = H, Cl, Br) [26]. The negative mode is important for the identification of halogenated POPs due to their high electron affinities. Reactions with O_2_^•−^ have also been shown to be structure-diagnostic, and this will be discussed in Section 3.4. It is also possible to generate Cl^−^ adducts by placing a vial of chloroform in the ion source, and this reaction appears to be selective towards polyhalogenated alkanes [67].

As the most popular GC–API technique, a number of publications have recently appeared that demonstrate its advantages over EI or CI. Analysis of hydroxypyrene (a PAH metabolite in human urine) using GC–APCI showed lower detection limits and a wider linear range than LC–MS/MS [52]. In the same vein, GC–APCI analysis resulted in >10-fold lower method detection limits for halogenated dioxins and furans in sediments, fish and fire debris as well as 9-nitrophenanthrene and 3-nitrophenanthrene in PM 2.5, as compared to using EI. GC–API techniques may also enable faster analysis. Unlike vacuum ion sources, API is inherently resilient to high flows of both nitrogen and helium carrier gases. For example, Di Lorenzo et al. demonstrated that PBDEs could be separated in less than 7 min and 15 min, respectively, with helium and nitrogen carrier gas [68]. Critical isomers could be separated using nitrogen, which is desirable in the face of looming shortages of helium, a non-renewable resource. A drawback of GC–APCI is increased ion suppression compared to EI. NTS practitioners should be cautious when using APCI to analyze compounds with a wide range of ionization energies. While preserving the sample information is a benefit, an interfering matrix could obfuscate unknown pollutants.

### 2.2. Atmospheric Pressure Photoionization (APPI)

APPI employs UV light from a Xe, Kr, or Ar lamp, to produce 8.4 eV, 10.1 eV and 11.2 eV photons, respectively, for ionization. A Kr lamp is commonly used because its longevity exceeds that of an Ar lamp while also providing energetic photons that are capable of ionizing a wider range of compounds than a Xe lamp. The formation of positive ions M^•+^ will occur if the photon energy exceeds the ionization energy of M, and akin to APCI, negative ions can form by electron attachment or through reactions with O_2_^•−^. Introduction of a suitable dopant (e.g., toluene, acetone, anisole, and chlorobenzene, etc.) whose IE is smaller than the photon energy can promote protonation and/or adduct formation with the analyte and significantly enhance ionization efficiency [42,69] and broaden the range of chemicals subject to APPI ionization. For example, neutral perfluoroalkyl and polyfluoroalkyl substances [59] cannot be directly photoionized into M^•+^. Instead, negative ions, including adducts with oxygen, are generated with the assistance of a dopant. In this case, the direct ionization of the dopant (D) results in the formation of D^•+^ radical cations, as well as free electrons that can promote negative ionization.

**Figure 1:**
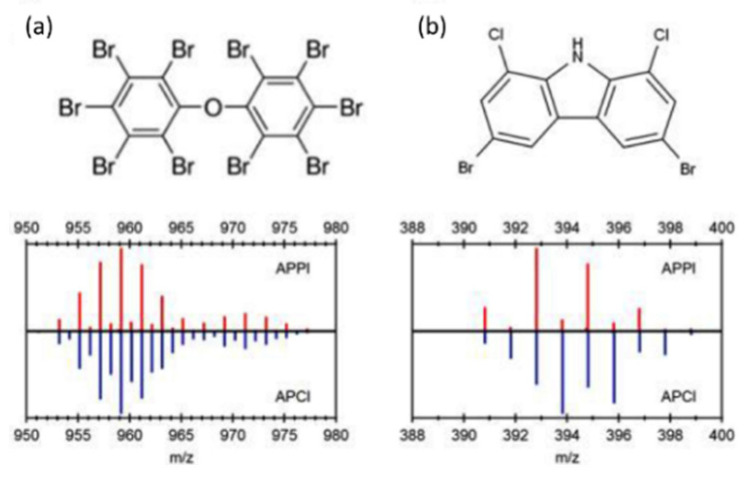
Partial mass spectra obtained using GC–APPI (**top**) and GC–APCI (**bottom**) for (**a**) decabromodiphenylether (BDE-209) and its ^13^C-labelled counterpart, and (**b**) 1,8-dibromo-2,6-dichloro-9H-carbazole. Reprinted from Di Lorenzo et al. [68]. Copyright 2019, with permission from Elsevier.

APPI is a more convenient way of selectively ionizing compounds using different photon energies compared to charge exchange reactions with different gases in APCI. It is desirable in some cases to exclude the ionization of H_2_O, which drives the formation of [M + H]^+^ rather than M^•+^ radical cations by charge exchange. Figure 1a shows the partial mass spectra of PBDE-209 and its ^13^C_12_-labelled analog obtained under both APPI (top) and APCI (bottom) conditions. Since H_2_O cannot be ionized by 10 eV photons, no subsequent protonation occurs, and only M^•+^ ions are observed in the APPI spectrum. In contrast, APCI results in a mixture of M^•+^ and [M + H]^+^ ions, increasing the complexity of the mass spectrum. This contrast is more evident for 1,8-dibromo-2,6-dichloro-9H-carbazole (Figure 1b), which belongs to an emerging class of POPs believed to occur as byproducts of halogenated indigo dyes [68]. [M + H]^+^ ions dominate the APCI spectrum because the presence of nitrogen increases the PA, but the APPI experiments produce M^•+^ ions only. The ability to control the types of (quasi)molecular ions being generated is an advantage of APPI because: (i) the complexity of the mass spectra is reduced; and (ii) ionization efficiency for a wide range of POPs is more uniform while also excluding potential interfering compounds whose IEs exceed 10 eV.

Excluding the ionization of compounds whose IEs exceed the photon energy of the lamp can also be a limitation. Obviously, the number of compounds that can be identified in such an experiment will decrease, and the use of dopants can also have the same effect. For example, a comprehensive comparison between APPI with and without dopants was performed for 75 EPA priority environmental pollutants. The study showed that the use of dopants increased the ionization efficiency for many compounds, but ultimately decreased the number of compounds detected [69].

### 2.3. Atmospheric Pressure Laser Ionization (APLI)

APLI is similar to resonance-enhanced multiphoton ionization (REMPI) [27], wherein two absorption steps are involved in ionization. The absorption of the first photon results in the formation of an excited molecule M*, which is subsequently ionized by a second photon to form M^•+^. This process is favorable if (i) the combined energies of the photons are resonant with the energy required for excitation and ionization, and (ii) the excited state M* is sufficiently long-lived to absorb a second photon. These two requirements are usually satisfied by π-electron-rich compounds [70] and most applications of APLI have focused on PAH and other aromatics (see Table 1). The high sensitivity of this technique allows sample dilution of up to 1000-fold, resulting in significantly decreased matrix interference, better separation and improved peak shape [64]. To expand the range of compounds that can be analyzed by APLI, Deibel et al. [58] introduced a series of APLI ionization labels to derivatize amines, alcohols and carboxylic acids. A recent comparison between APPI and APLI revealed that APPI was able to ionize the widest range of analytes (66/77) and halogenated aromatics were much more readily ionized by APPI than by APLI [69].

### 2.4. Electrospray Ionization (ESI)

ESI involves the creation of ions from charged droplets. In LC–MS, the column effluent is delivered through a charged capillary. Gas phase ions are produced as the solvent molecules are stripped away with the aid of a flow of nitrogen. ESI produces a wide range of ions that originate from the solvent itself, including protonated molecules and dimers, (sodium and potassium) cationized adducts and cluster ions. In the negative ion mode, ESI produces deprotonated molecules, as well as adducts and clusters bridged by common anions present in the solvent or mobile phase, such as Cl^−^ or formate. The electrosprayed droplet–ions can also serve as vehicles for protons and other reagent ions that may ionize solid, liquid or gaseous samples. For example, in desorption electrospray ionization (DESI), charged droplets are directed towards a solid, extracting molecules from the surface while transferring charge from the ionized solvent to the analyte. At the same time, the droplet bounces from the surface, propelling the analyte ions towards the entrance of the MS for analysis. Similarly, in extractive electrospray ionization (EESI), the charged droplets collide with and ionize a secondary aerosol spray. In principle, the same approach could be used to ionize gaseous molecules exiting a GC column, but to date, GC–ESI has only been attempted by one group [65,66]. The results of Cha et al. [65] showed that GC–ESI could achieve linearity, repeatability, robustness and detection limits comparable to standard GC–MS and LC–MS methods. Even non-polar PAHs could be ionized by GC–ESI, and protonated molecules [M + H]^+^ dominated their mass spectra. GC–ESI is a relatively unexplored means of introducing reagent ions, such as metal cations and anions [71], that would not be possible using GC–APCI. Whether such ion chemistry will be useful for structure elucidation is a question that deserves more attention.

### 2.5. Penning Ionization (PI)

Direct Analysis in Real-Time (DART) was first introduced by Cody and Laramee [32,72]. Penning ionization is initiated by glow discharge of a gas (commonly helium), resulting in neutral atoms in a metastable excited state. The internal energy of metastable helium (19.8 eV) exceeds the ionization energies of most common atmospheric gases. While Penning ionization may result in the formation of radical cations M^•+^, water from the ambient environment will also ionize, self-protonate and form cluster ions. Akin to APCI, the formation of protonated molecules [M + H]^+^ will occur when M has a higher PA than H_2_O and its cluster ions. In the vast majority of applications, DART is directly coupled with a mass spectrometer [32]. Penning ionization has also been employed for ionization of GC effluent by Moore et al. [73] and later by Cody et al. [32]. In general, the appearance of mass spectra obtained by GC–DART are similar to those obtained by GC–APCI.

### 2.6. Dielectric Barrier Discharge Ionization (DBDI)

The ionization process is initiated by a dielectric barrier discharge, which involves the creation of a low temperature plasma (LTP, ~30 °C) that is ignited when a potential is applied between two electrodes separated by a dielectric material. It was first introduced as a GC–MS ion source by Nørgaard and coworkers in 2013 [74]. Similar to DART, LTP also produces M^•+^ and [M + H]^+^ ions in the positive mode, and [M − H]^−^ and M^•−^ ions in the negative mode. As a proof of concept, Nørgaard et al. demonstrated that 20 common indoor VOCs, including alkanes, alkenes, alcohols, aromatic compounds, aldehydes, PAHs, phenols, and terpene alcohols, could be detected using this approach [74]. In 2017 [75], Hagenhoff and coworkers developed a similar DBDI source, albeit with electrodes configured in a different geometry. The exquisite sensitivity of the approach enabled the detection of femtogram levels of 28 pesticides and 14 illicit drugs. Their ion source also showed promise when applied to NTS:GC–DBD was used to screen semifluorinated n-alkanes (SFAs) in ski wax samples [76]: SFAs with carbon numbers of 26, 28, 30, and 32 were tentatively identified and 1-(perfluorooctyl)-hexadecane confirmed with an authentic standard.

## 3. Strategies to Identify Unknowns by GC–API

### 3.1. Multidimensional Chromatography

Recent interlaboratory studies of NTS methods suggest that a combination of complementary chromatography methods and ionization sources is essential to detect and identify all compounds present in a sample [7,77]. For example, the identification of approximately 1500 organic pollutants in surface and groundwater surrounding a solid-waste treatment plant required the use of multiple techniques, including GC–(EI)TOF, GC–(APCI)QTOF, LC–(ESI)QTOF and LC–(ESI)QqQ [78]. There is also a growing interest in developing multidimensional separation techniques that involve multiple separation stages in a single experiment.

**Figure 2:**
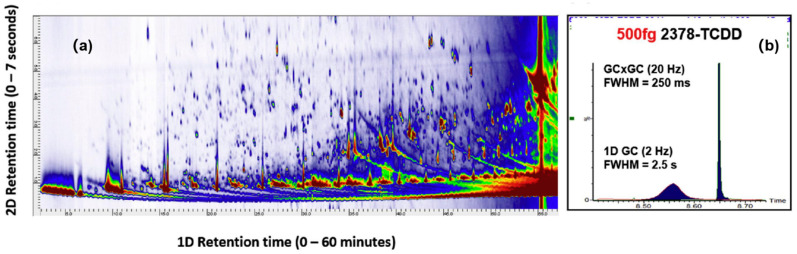
(**a**) Comprehensive two-dimensional gas chromatography (GC×GC) affords unparalleled separation of organic pollutants from in plasma; (**b**) Modulated gas chromatogram obtained from 2,3,7,8-tetrachlorodibenzo-*p*-dioxin, resulting in 10-fold signal-to-noise (S/N) enhancement. Similar S/N enhancement can be achieved using either thermal or flow modulation. Reprinted from Ref. [80]. Copyright 2020, with permission from Elsevier.

GC×GC can significantly increase the number of identifiable compounds in a sample compared to using single-dimension GC–MS. Ballesteros-Gómez et al. [79] were the first combine GC×GC with APCI and it has since been applied to characterizing plasma [80] and household dust [7]. The contour plot in Figure 2a was obtained from a pooled sample of plasma and it may serve to demonstrate the separation power of GC×GC. It is evident from the plot that many compounds can be separated by the second dimension column that would otherwise coelute in the first dimension. In the interlaboratory study led by Roskowski et al. [7], both GC×GC–APCI and GC×GC–EI were used to tentatively identify >500 compounds, representing a large fraction of the total number of compounds reported by all participants. It is because the improved separation resulted in the collection of higher quality (CID) mass spectra used for identification. Separation is not the only benefit afforded by modulating effluent from the primary column. As shown in Figure 2b, the width of a GC×GC peak is very narrow and more intense compared to one collected using a single-dimension GC. Patterson et al. [81] pioneered the approach called cryogenic zone compression. Their result showed that it could improve the detection limits for trace level contaminants such as PCDDs. When coupled with GC–API and a full-scanning, high-resolution mass spectrometer, the technique could potentially enable the identification of unknown contaminants with volume-limited samples, such as dried blood spots [80].

Most GC×GC instruments employ cryogenic modulation, whereby the primary column effluent is trapped by a flow of cooled nitrogen gas, and then reinjected into the secondary column by a pulse of heated nitrogen. GC×GC may alternatively be accomplished using a flow modulator, which converts primary peaks to secondary peaks using pulses of gas flow delivered using one or more valves without cryogens. Valve-based modulators do not technically zone compress (i.e., focus) the GC effluent, but the width of the secondary peak may still be controlled by the flow, which can exceed the primary flow by 10 times or more. Such a configuration is not compatible with conventional EI and CI ion sources. In contrast, flows in excess of 100 mL/min are well suited to the conditions of GC–API. A multimode flow modulator designed by J.V. Seeley [82] was adapted to a GC–APCI instrument, resulting in a significant enhancement in sensitivity [83].

**Figure 3:**
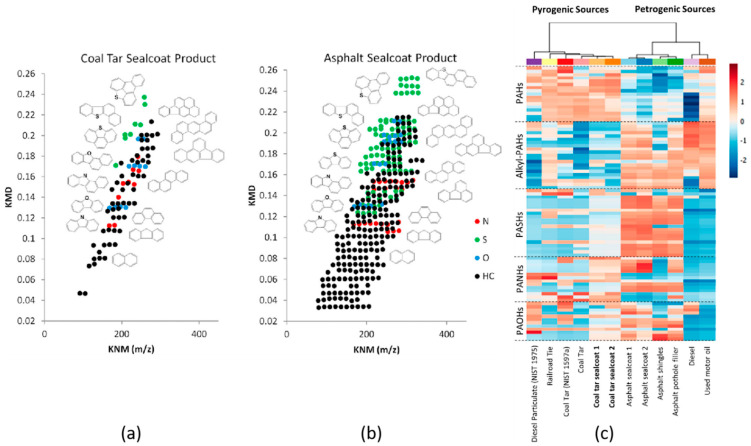
Kendrick mass defect (KMD) plots of (**a**) a coal tar sealcoat product, and (**b**) an asphalt sealcoat product. (**c**) Two-dimensional hierarchical cluster analysis with heat map plot of various environmental PAH sources. The colors red and blue represent the most intense and least intense relative abundance values (log scale). The relative abundances in the heat map plot are presented as an average of three replicates. Reproduced from Bowman et al. [84] with permission. Copyright 2019 American Chemical Society.

The profile of compounds detected by GC×GC–API, sometimes referred to as a “chemical fingerprint”, has proven to be vital for identifying potential sources of pollution. For example, Bowman et al. [84] used GC×GC–APCI to characterize the components of coal tar-based sealcoat products in comparison to those in other sources of polycyclic aromatic compounds (PACs). The results revealed that there was a clear difference in the composition of PACs across different types of sealcoat products and sources of PAHs. Figure 3a,b displays the Kendrick mass defect plots obtained from coal tar and asphalt sealcoat products, respectively. They have shown that the petrogenic asphalt sealcoat is characterized by a greater range of alkylated PAH, compared to the pyrogenic coal tar sealcoat. Individual components were also identified by accurate mass measurements in combination with first dimension retention indices. Hierarchical clustering analysis led to the identification of signature compounds that could distinguish coal tar from other pyrogenic sources of PAH pollution, such as creosote (from a railroad tie), and diesel particulate.

### 3.2. Data-Independent Identification

In NTS, an important benefit afforded by “soft ionization techniques like GC–APCI is the abundant formation of (quasi)molecular ions whose accurate mass can determine an unknown’s elemental composition. However, a major weakness is the loss of structure-diagnostic fragmentation normally obtained by EI. Instead, the structural identity of newly discovered contaminants is typically achieved using tandem mass spectrometric techniques, such as collision-induced dissociation (CID). Two strategies have emerged for automated collection of CID mass spectra during GC (×GC) separation, viz. data-dependent acquisition and data-independent acquisition (DIA).

**Figure 4:**
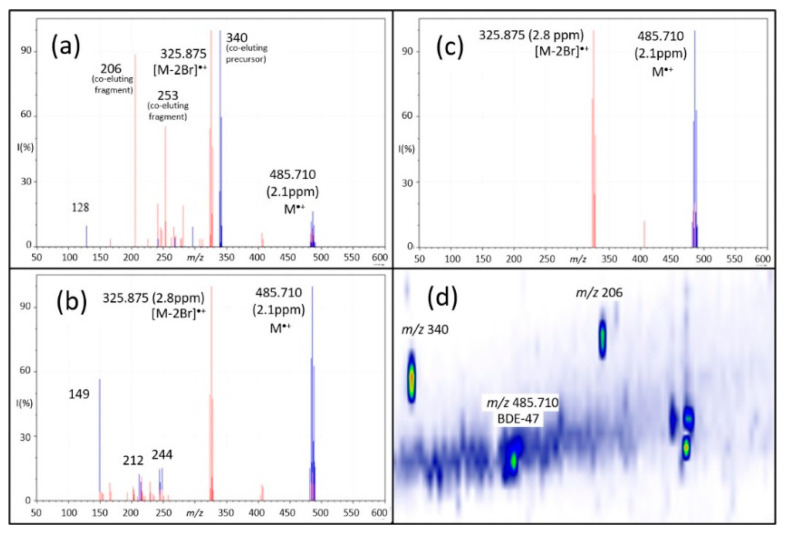
Comparison of (**a**) GC–DIA, (**b**) GC×GC–DIA, and (**c**) deconvolved GC–SQDIA spectra of a tetrabromodiphenyl ether. In all spectra, the low-energy channel is shown in blue and the high-energy channel is shown in red. (**d**) GC×GC–DIA chromatogram showing separation of compounds that coeluted in one-dimensional GC–DIA (as seen in (**a**)). Greater separation in both GC×GC–DIA and GC–SQDIA resulted in spectra with fewer interferences than those obtained using GC–DIA; this separation can be obtained chromatographically, as in GC×GC, or utilizing the quadrupole, as in SQDIA. Adapted with permission from Schreckenbach et al. [20]. Copyright 2021 American Chemical Society.

Data-independent acquisition [85] was first introduced for the analysis of peptide mixtures in combination with LC–MS separation. It was only recently applied to GC–APCI for suspect screening and NTS of organic pollutants [20]. Compared to data-dependent acquisition, which selects ions based on predefined criteria such as mass, intensity or isotopic ratios, the DIA approach enables the automated, unbiased selection and acquisition of the precursor and CID mass spectra of all ions detected in the sample [20]. By cycling between low- and high-collision energy, precursor and product ions can be identified by changes in their intensities: precursor ions will decrease in intensity when subjected to high collision energy, whereas the opposite is true for fragment ions. A computer algorithm then deconvolutes the CID mass spectrum of each compound by grouping together precursor and product ions that share the same GC retention time. A drawback of this approach is its inability to deconvolute CID mass spectra of compounds that coelute. For example, Figure 4a shows the CID mass spectrum of the flame retardant PBDE-47 that was collected using DIA during a short 15 min GC separation [20]. Numerous interfering peaks (*m/z* 206, 253 and 340) can be observed that are absent when a longer GC×GC separation was performed, see Figure 4c. However, it is not always possible to separate coeluting compounds, and in this case, the time requirement for the GC×GC separation exceeded that of the single-dimension experiment by four-fold [20]!

One way to obtain a better-quality CID mass spectrum is to use the quadrupole analyzer to cycle through narrow isolation windows (typically c. 20~50 amu segments) and sequentially subjecting the selected ions to CID. For example, a mass spectrum with a range of 20~1000 amu can be subdivided into 49 segments or *swaths*, which are isolated and fragmented. This was pioneered by Gillet et al. [86], who coined the approach Sequential Windowed Acquisition of all Theoretical fragment ion–Mass Spectra (SWATH–MS). Scanning quadrupole DIA (SQDIA) is closely related to SWATH–MS, but instead of cycling the quadrupole between *swaths*, it is scanned continuously across the mass range [87]. This significantly reduces coeluting interferences in the resulting CID mass spectra, as illustrated by comparing the DIA and SQDIA mass spectra obtained for PBDE-47 shown in Figure 4a,b, respectively. SQDIA can produce CID mass spectra without sacrificing the time required for a more rigorous chromatographic separation. The gain in selectivity and speed provided by SQDIA, however, also comes with a cost in sensitivity: while the quadrupole is scanning, only a small subset of the ions proportional to the isolation window (IW) are transmitted to the detector and the remaining ions are lost. The theoretical transmission can be calculated using the equation: IW (amu)/mass range (amu) = transmission (%).

GC–SQDIA has been used to screen 2542 suspected organic contaminants listed in the AMAP (Arctic Monitoring and Assessment Programme) 2016 Chemicals of Emerging Arctic Concern in a dust sample collected from an electronics recycling facility. The procedure for structure assignment involved comparing the measured mass of the (quasi)molecular ions with the theoretical masses of the 2542 suspected pollutants. Then, a software tool (MassFragment [88]) was used to predict the possible fragments from each of the structures in the library. The structure that produced the greatest number of fragments matching with those observed in the CID mass spectrum was then assigned. In this case, the software tool was part of a commercial software package (UNIFI [89]), but the same structure assignment procedure could be applied using open source software such as: MetFrag [90]; CSI (Compound Structure Identification): FingerID [91]; CFM (competitive fragmentation modeling)-ID [92]; and QCEIMS [93]. A more detailed discussion on the prediction of mass spectra will be presented in Section 3.6. The results of this suspect screening experiment showed that SQDIA significantly lowers the rate of false structure assignment.

Ion mobility can also be used to disentangle the CID mass spectra of coeluting compounds [94]. One requirement of this approach is that compounds that coelute from the GC column must be separable according to their mobility, which can be characterized by an ion’s collisional cross section (CCS). At elevated pressures, non-reactive collisions impede the ions akin to an aircraft in flight such that large, bulky ions tend to have larger CCS values and lower mobilities than small, compact ions. CCS values can also be used as confirmatory evidence of a structure assignment [21]. Lipok et al. were the first to combine GC×GC–APCI with ion mobility–mass spectrometry. They used an in-house database consisting of 800 CCS values to screen for drug-like compounds and pesticides [21]. Recently, Olanrewaju et al. [95] have analyzed PAHs and related petroleum hydrocarbons in crude oil using a trapped ion mobility–mass spectrometer hyphenated with GC. In this study, the identification of isomeric PAHs and related unknown aromatic hydrocarbons was accomplished with the aid of CCS measurements. The results also raise the intriguing possibility of separating small contaminant molecules by ion mobility alone. For example, the difference in collisional cross sections (∆ CCS) of the isomers triphenylene and chrysene is only ~2 Å^2^. Using the formula R = CCS/∆CCS, it is anticipated that an ion mobility resolution of ~73 would be sufficient to resolve isomers that are closely eluted by GC. Ion mobility–mass spectrometers capable of R > 200 are now commercially available [96], but it has yet to be shown that this separation is fast enough to be compatible with GC (×GC).

### 3.3. Evaluating Confidence in Structure Assignments

According to Schymanski et al. [77], structure assignments based on accurate mass and isotopic measurements of their (quasi)molecular ions may be considered tentative at best, corresponding to structure confidence levels 4 and 5 on their 5-level scale. The highest confidence score, level 1, requires confirmatory evidence obtained using authentic standards. While a meaningful study of the occurrence and fate of organic pollutants will require authentic standards [5], it is also recognized that this is not always practical at the earliest stage of a contaminant’s discovery. In the absence of authentic standards, acquiring complementary information such as CID mass spectra, retention time(s) and CCS can increase the confidence in a tentative identification.

The time-honored approach to identifying an unknown pollutant involves comparing its EI mass spectrum with those compiled in spectral libraries. There are databases containing hundreds of thousands of EI spectra (e.g., the NIST Mass Spectral Library), but equivalent libraries compiling CID mass spectra are orders of magnitude smaller. For example, Mesihää et al. [44] have developed an in-house GC–APCI–QTOFMS library that includes 29 psychoactive substances. However, creating spectral libraries is time-consuming and costly. To bridge this gap, practitioners of GC–API can take advantage of workflows that were originally conceived for LC–MS. Instead of relying on spectral libraries, one could search structure libraries (e.g., PubChem and ChemSpider) and then compare the experimental CID mass spectra with those predicted by in silico methods [97]. This approach typically involves comparing the measured mass of the (quasi)molecular ions with the theoretical masses of all compounds in the structure library. Then rules-based or combinatorial fragmentation predictors are used to predict the possible fragments from each of the structures in the library whose molecular ions fall within a preselected mass range (usually 1–5 ppm) of the experimental mass. The structure that produces the greatest number of fragments that match with those observed in the CID mass spectrum is then assigned.

Su et al. [98] have suggested a modified version of the confidence scale proposed by Schymanski et al. [77]. Their approach hinges on comparing results obtained by GC–APCI-MS and GC–EI–MS, taking advantage of both structural and spectral libraries [99]. Briefly, the criteria for a level 3 identification are: (i) a compound’s EI spectrum must match that of a library spectrum with a match factor >700; and (ii) the mass of the compound’s (quasi)molecular ion peak must fall within 5 ppm of the theoretical mass. Confidence in the proposed structure increases to level 2 when complementary evidence, such as retention index or CCS, is used. In the absence of a good quality spectral library match, a structure library search can also be used in combination with careful interpretation of the CID mass spectrum. This requires a firm understanding of the dissociation chemistry of organic ions and their reactivity with gas molecules used in the ion source and collision cell. Ultimately, a tentative identification must be confirmed with an authentic standard.

### 3.4. Ion-Molecule Reactions for Separation and Structural Elucidation

**Figure 5:**
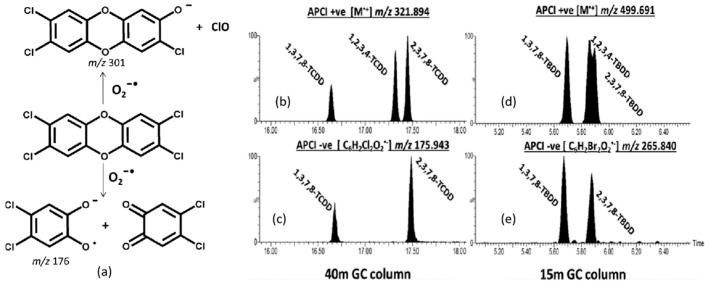
(**a**) Scheme of reaction between 2,3,7,8-TCDD and O_2_^−•^; (**b**–**e**) APCI− spectra of 2,3,7,8-TCDD, 2,3,7,8-TBDD, and 2,3 Br-7,8 Cl-DD. Ether cleavage products (ECPs) are observed at *m/z* 176 and 266 for the 2,3,7,8-TCDD and 2,3,7,8-TBDD species. Both of these ECPs are observed for the 2,3 Br-7,8 Cl-DD. Reproduced with permission from Ref. [103]. Copyright 2016 American Chemical Society.

The conditions of the GC–APCI ion source can promote ion–molecule reactions that are structure-diagnostic and, in some cases, can differentiate between toxic and non-toxic isomers. A prime example is the reaction between dioxygen and (mixed) halogenated dibenzo-p-dioxins in the negative ion mode. Quasimolecular ions [M − Cl + O]^−^ are generated by reactions between the analyte molecules and O_2_^•−^. Mitchum and Korfmacher et al. [100,101,102] showed that the reaction between 2,3,7,8-tetrachlorodibenzo-*p*-dioxin (2,3,7,8-TCDD) and O_2_^•−^ also results in cleavage at the ether bonds of 2,3,7,8-TCDD, as shown in Figure 5a. This specific reaction was shown to distinguish 2,3,7,8-TCDD from many other common interfering species, including its isomers. For example, the negative ion APCI mass spectrum of 2,3,7,8-TCDD displays an intense peak at *m/z* 176, corresponding to the ether cleavage product shown in Figure 5a. In contrast, 1,2,3,4-TCDD cannot produce a peak at *m/z* 176 because all four of its chlorine atoms are present on the same ring. As shown in Figure 5b–e, this difference can be exploited to reduce the burden on GC to separate TCDD isomers.

The ubiquity of brominated flame retardants in everyday household items increases the likelihood that PBDDs will be formed during (accidental) fires [104]. Highly brominated contaminants, including the tetrabromoodibenzop-dioxins (TBDDs), are challenging to analyze by GC–MS because of their thermal lability. To minimize thermal decomposition during chromatographic separation, a relatively short (~15 m) GC column is used along with a relatively thin (0.1 μm) stationary phase. Therefore, separating toxic from non-toxic isomers is a major challenge that cannot be solved using GC alone, as witnessed by the coeluting isomers 2,3,7,8-TBDD (toxic) and 1,2,3,4-TBDD (less toxic) in Figure 5d. Fernando et al. [103] showed that ion–molecule reactions with oxygen could be exploited to separate the coeluting isomers because 2,3,7,8-TBDD reacts with oxygen to produce the ether cleavage product C_6_H_2_BrO_2_^•−^ (*m/z* 265.840), whereas 1,2,3,4-TBDD cannot. When applied to samples collected from a major industrial fire, this ion chemistry could differentiate isomers of PXDDs (where X = Cl, Br) that would not be feasible using EI.

Structure-diagnostic reactions have also been observed in the positive ion mode^68^. For example, Di Lorenzo et al. [68] observed that the PBDE flame retardants can undergo isomer-specific photooxidation in a GC–APPI source, viz. that PBDE-71 produces an [M − Br + O]^+^ ion in its APPI mass spectrum that is absent in that of PBDE-49. EPA method 1614 requires the separation of isomers PBDE-49 and PBDE-71, but the observation that GC–APPI can differentiate isomeric PBDEs raises the possibility that this requirement may be relaxed. The group of R. G. Cooks [105] has shown that corona discharge under solvent-free conditions can promote oxidation reactions of alkanes that would otherwise require catalysis. Megson et al. [106] have also observed similar reactions under GC–APCI conditions: the ubiquitous plasticizer and flame retardant tricresyl phosphate (TCP) undergoes an uncatalyzed oxidative transformation into the metabolite 2-(ortho-cresyl)-4*H*-1,3,2-benzodioxa-phosphoran-2-one (CBDP), which is responsible for the neurotoxicity of TCP. This ion–molecule reaction is specific to ortho-substituted triaryl phosphates, which are toxic, unlike the non-toxic meta- and para-substituted isomers. The reaction also mirrors the microsome/enzyme-promoted transformation that occurs in vivo. There is currently little fundamental understanding of this reactivity, but the implications for NTS are significant: substituted aryl phosphates are widespread environmental contaminants and an indoor dust sample may contain hundreds of (unknown) homologs. GC–APCI could potentially be used to identify the neurotoxic ortho-substituted isomers in such a mixture selectively.

### 3.5. Retrospective Analysis and Compound Discovery

**Figure 6:**
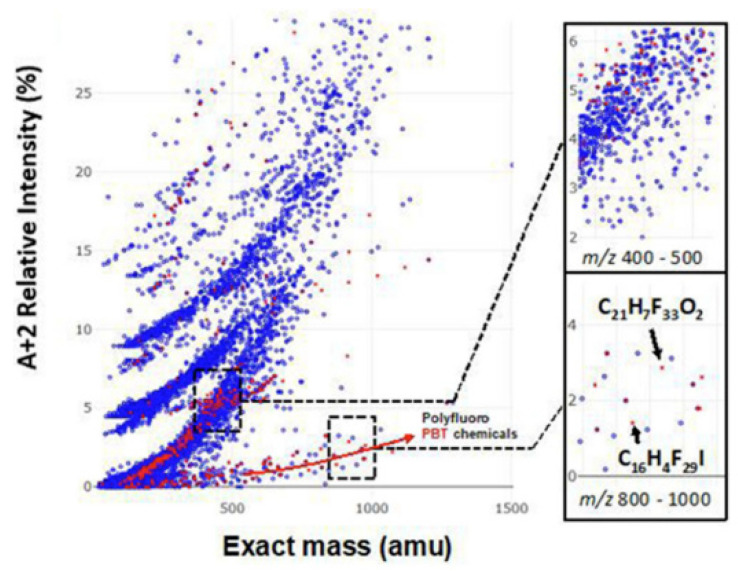
Distribution of 610 prioritized persistent bioaccumulative compounds (in red) and commercial chemicals (in blue) from the North American chemical inventories in the compositional spaces defined by *m/z* of the molecular ion and the ratio of its isotopic peaks (A + 2): A, where “A” is the most intense isotopic peak. Adapted from Zhang et al. [41]. Copyright 2019, with permission from Elsevier.

A key advantage of all HRMS techniques is that full scan results can be digitally archived and exploited retrospectively. Lai et al. [107] recently developed a prescreening and identification workflow implemented as an R package to support regulatory environmental monitoring. One of the challenges of retrospective analysis is developing a strategy to recognize pollutants from among the many thousands of chemicals detected by HRMS. Zhang et al. [41] recently developed an approach to identify unknown persistent and bioaccumulative organics using mass spectrometry and applied this approach using GC–APCI. Most POPs contain three or more Cl or Br atoms, making them easy to recognize based on their isotope patterns. Even polyfluorinated compounds can be recognized based on a relatively weak ^13^C-isotopic peak compared to non-fluorinated compounds, see Figure 6.

Data collected using GC–APCI is ideally suited for retrospective analysis because the molecular ion is preserved. Zhang et al. [41] identified 191 isotopic clusters from a housedust standard reference material using their prioritization strategy. The identified chemicals included PCBs, agricultural drug residues, polychlorinated and polybrominated diphenyl ethers and other brominated flame retardants. Previously unknown chlorofluoro flame retardants were also discovered in this study, including thermal decomposition products of 2,3,4,5-Tetrachloro-6-((3-(trideca-fluorohexyl)sulfonyloxy)phenylaminocarbonyl)benzoic acid.

### 3.6. Computational Tools to Predict Mass Spectra

Libraries of experimental CID mass spectra are much smaller than those compiled for EI. To bridge this gap, novel computational tools have emerged to predict CID spectra. These tools may be broadly classified into three types, reviewed by Scheubert et al. [97], and have been widely used to predict CID mass spectra collected during LC–MS experiments. The most common type utilizes either a rules-based or a combinatorial approach to predict the fragmentation of an ion. Examples include MetFrag [90], MassFragment [108] and Mass Frontier [109]. These methods do not technically predict the spectrum, but rather identify the number of experimentally observed peaks that can be explained by a given structure. This approach is popularly used for suspect screening and structural database searching, but one limitation is the fact that an unknown’s structure must be present in the database in order for a search to be successful. Another limitation is that these methods do not predict peak intensities, which could be used to assign a structure more reliably. In contrast, recently developed machine learning methods, such as CFM-ID [92], can predict whole mass spectra, including relative intensities, but their accuracy depends on the size of the training set used. This is problematic for GC–API because there are few experimental CID spectra available. CSI: FingerID [91] combines fragmentation tree computation and machine learning to predict the molecular fingerprint of the unknown compounds. Another spectra prediction tool is based on computational chemistry. Quantum chemical electron ionization mass spectrometry (QCEIMS) [93] employs semiempirical quantum mechanical and/or density functional theory methods, and Born-Oppenheimer molecular dynamics to predict the dissociation behavior of radical cations. While this approach is the most accurate way to predict a mass spectrum, it is also the most resource-demanding, requiring approximately 1000 core hours to compute the spectrum of a small molecule pollutant.

**Figure 7:**
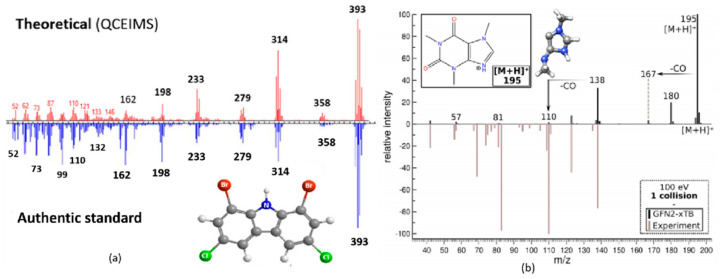
(**a**) EI mass spectrum calculated by QCEIMS and experimental EI mass spectrum of 3,6-dichloro-1,8-dibromo-carbazole. Reproduced from Ref. [110]. Copyright 2021 American Chemical Society. (**b**) calculated spectrum and 40 eV literature spectrum of the most populated caffeine protomer at 600 K, single collision at 100 eV in the laboratory frame. Reprinted from Ref. [111]. Copyright 2021 American Chemical Society.

CSI: Finger ID, CFM-ID and QCEIMS have all been used to predict CID spectra and guide the interpretation of GC–MS data. CSI: Finger ID was used by Larson et al. to identify products of lignin pyrolysis [42]. QCEIMS was evaluated by Schreckenbach et al. [110] to predict the mass spectra of selected halogenated and organophosphorus flame retardants. While QCEIMS is designed for EI spectral prediction, it can also be informative when predicting the CID mass spectra of ions M^•+^ generated by charge exchange (GC–APCI) or photoionization (GC–APPI). This is because the unimolecular dissociation behavior of an ion is largely determined by the potential energy surface that does not depend on the ionization technique. The results showed that QCEIMS predicted the mass spectra of 35 organic pollutants as accurately as the less computationally demanding CFM-ID method. QCEIMS is best suited for compounds that are truly unknown and thus not present in any library or training set. For example, QCEIMS accurately predicted the EI mass spectrum of the dioxin-like compound 1,8-dibromo-3,6-dichlorocarbazole (shown in Figure 7), an emerging contaminant of the Laurentian Great Lakes. A recent study [111] reports on the development of QCxMS, which extends QCEIMS for the prediction of both EI and CID spectra prediction. A test with six standards showed the calculated data was in reasonable agreement with the experiment (see Figure 7b). These methods can help guide the analyst in selecting authentic standards.

## 4. Summary and Outlook

GC–API techniques generally minimize fragmentation and preserve the (quasi)molecular ion, resulting in simplified mass spectra and improved detection limits. It is relatively facile to modify an existing LC–MS instrument and increase its analytical power. The conditions of the ion source are compatible with high flows that enable faster analysis as well as ion–molecule reactions that can aid in structure analysis. Moreover, spectacular advances in mass spectrometry have led to the development of novel mass analyzers and ion mobility–mass spectrometers that have not previously been coupled to GC, enlarging the scope of GC–MS analysis. It is now possible to use techniques and workflows, such as data-independent acquisition, to significantly decrease the false-positive rate for unknown structure assignments.

GC–API is already a complementary to EI and it may eventually supplant it as the de facto GC–MS ion source used to identify unknowns, but to achieve this a number of challenges will need to be surmounted. First, the absence of libraries of CID mass spectra is the most obvious challenge. However, EI mass spectra can still inform the interpretation of GC–API experiments, considering both GC–API and EI can produce the same types of ions. The dissociation chemistry of a radical cation M^•+^ is only partly dependent on how it is formed, and it is likely that the low-energy reactions will be observed in both experiments. There are also emerging computational techniques that promise to predict CID mass spectra reliably and construct in silico spectral libraries. Second, the ion chemistry that occurs under GC–API conditions has not been fully exploited and in some cases, it is not completely understood. Recent studies have shown that uncatalyzed reactions occurring in the ionization source can aid in structure analysis, e. g. oxygen or nitrogen insertion or displacement reactions that can differentiate toxic from non-toxic isomers [105]. To fully exploit these reactions, their mechanisms will need to be revealed by an integrated experimental and computational approach. Finally, GC–API, when coupled with HRMS, ion mobility and other sophisticated analyzers, is capable of detecting tens of thousands of chemical compounds. New strategies are required for retrospective analysis, annotation of mass spectra and to prioritize the identification of environmentally relevant compounds.

## Figures and Tables

**Table 1:** Recent studies that employ GC–API–MS techniques for (non)targeted analysis of environmental pollutants.

	Sample	Detector	(Non)Targeted Chemicals	Method Merits	Ref.
APCI	Food packaging materials	QTOF, HP-5MS	Acrylic adhesives including 2-methyl-1,2-thiazol-3(2*H*)-one, 5-chloro-2-methyl-1,2-thiazol-3(2*H*)-one and 1,2-benzothiazol-3(2*H*)-one		[36]
APCI	Polyurethane foam disks (PUFs), food, and marine samples	Xevo TQ-S QQQ	Hexabromocyclododecane	IDL: 0.10 pg/μL RSD: <7%	[37]
APCI	Surface water, groundwater, wastewater		Pesticides, polycyclic aromatic hydrocarbons (PAHs), polychlorinated biphenyls (PCBs), Polybrominated diphenyl ethers (PBDEs), fragrances, musks, antimicrobials, insect repellents, UV filters, polychloronaphthalenes (PCNs)		[38]
APCI	Indoor air sample	QTOF, HP-1-MS	volatile, intermediate-volatility, and semivolatile organic compound	LOD: 10~100 ppq	[39]
APCI	Chinese mitten crab food webs	Xevo TQ-XS QQQ, DB-5MS	PCBs (mono-to deca-) and polychlorinated dibenzo-p-dioxins/dibenzofurans (PCDD/Fs)	RSD: PCBs: 3.4%~15.5%; PCDD/Fs: 1.7%~7.9% LOD: PCBs: 0.021~0.150 pg/mL; PCDD/Fs: 0.051~0.237 pg/mL	[40]
APCI	Standard Reference Material (SRM 2585) of household dust	QTOF	191 POPs including PCBs and agricultural drug residues, such as chlordane and degradation products of DDT and Fentichlor, polychlorinated and polybrominated diphenyl ethers (PCDEs and PBDEs), other brominated flame retardants such as tetrabromobisphenol A (TBBPA) and bis(2-ethylhexyl) tetrabromophthalate (BEHTBP), chlorine-containing organophosphate flame retardants tris(1,3-dichloro-2-propyl)phosphate (2 isomers), tris(2-chloroisopropyl)phosphate and tris(2-chloroethyl)phosphate		[41]
APCI	Electronic waste dust	Q-TOF DB5-HT	52 brominated, chlorinated, and organophosphorus compounds identified by suspect screening; 15 unique elemental compositions identified using NTS with 17 chemicals confirmed using standards		[20]
APCI	Low sulfonate lignin	Q-TOF TOF	59 lignin pyrolysis products were positively identified, with 10 chemicals confirmed using standards		[42]
APCI	Urine Blood	QTOF DB- 5MS	Illicit psychostimulant drugs		[43,44]
APGC	Low-temperature coal tar sample and its distillation products	TQ-S DB-35 MS	Phenolic compounds (phenols, indanols, naphthols, and benzenediols)		[45]
APGC	Human serums	Xevo TQ-S	Organochlorine pesticides (OCPs) and PCBs	RSD: <15%	[46]
APCI	Urine samples	QTOF	α-pyrrolidinovalerophenone metabolites		[47]
APGC	Food	FT-ICR, Rtx-1614	Halogenated flame retardants (HFRs)	Recovery: 59~115%; RSD: 5–15%; IQL: 1~5 pg/g; MQL: 0.002~0.04 ng/g	[48]
APCI	Urine	QQQ, HP Ultra 1	Exogenous androgenic anabolic steroids	RSD: 15–25% Most LOD: below 0.5 ng/ mL	[49]
APGC	Seal and egg samples	Xevo TQ-S QQQ, Rtx-1614	PBDEs, their methoxylated derivatives (MeO-PBDEs) and other emerging (brominated flame retardants) BFRs	RSD: <1. IDL: emerging BFRs, BDE 209 and MeO-PBDEs mixtures: 0.075~0.1 pg/µL; Br1–9 PBDEs mixtures: 0.625~6.25 pg/µL	[50]
APGC	Air fine particulate matter (PM 2.5)	Xevo TQ-S QQQ	Nitro-polyaromatic hydrocarbons	IDL: (0.20~2.18 pg/mL MDL: 0.001~0.015 pg/m^3^; Recovery: 70%~120%	[51]
APGC	Urine samples	Xevo G2-XS QTOF, DB-17+ custom MXT	1-Hydroxypyrene, 3-hydroxyphenanthrene, 9-hydroxyfluorene	1-Hydroxypyrene LOD: 0.64 ng/L, LOQ 2.16 ng/L; average CV: 11.5%	[52]
APGC	Simulated burn study samples (household and electronics), Particulate matter coating the firefighter’s helmets	Xevo TQ-S, Rtx Dioxin-2	Polyhalogenated dibenzo-p-dioxins/dibenzofurans (PXDD/Fs) and polybrominated dibenzo-p-dioxins/dibenzofurans (PBDD/Fs)	total levels of each halogenated homologue group: parts per billion	[53]
APGC	Fish, dust	Xevo TQ-S, Rtx Dioxin-2		Soil: MDL: 0.15~1.4 pg/g, RSD < 11% Fish: 0.21~2.0 pg/g, RSD < 33%	[54]
APGC	Food and feed	Orbitrap, DB-5MS	Polychlorinated dioxins and polychlorinated biphenyls	S/N: 753 for 40 fg on column Average RSD: 9.8%	[55]
APCI	Dust	Xevo G2-XS qTOF, DB-5 HT	40 PBDEs and 25 emerging HFRs	LOD: HFRs: 0.65 (0.016~9.1) pg/ μL; PBDE: 0.17 (0.0123~2.5) pg μL	[56]
APPI	Drug solutions	HR-LTQ Orbitrap, SLB-5 ms	Triazines and organophosphorus pesticides, PAHs, Drugs (diazepam and methadone)	Pesticide: average 3 pg/mL PAH: 0.1 pg/mL Drugs: average 30 pg/mL	[57]
APPI	Derivazation	oaTOF	Amines, alcohols, carboxylic acids	LOD: pmol~attmol	[58]
APPI	River water, tap water	HRMS (Q-Orbitrap)	fluorotelomer olefins (FTOs), fluorotelomer alcohols (FTOHs), fluoroctanesulfonamides (FOSAs) and sulfonamidoethanols (FOSEs)	LOD: 0.02–15 ng/L; RSD% < 11	[59]
APPI	Fruit and vegetable samples	QTOF	416 pesticides 416 pesticides		[60]
APLI	Human urine	TOF, DB-35	*Trans*-*anti*-benzo[*a*]pyrene-tetraol (BaP-tetraol) (PAH biomarker)	IOD of 0.5 fg	[61]
APLI	Rocks	HR TOF, RXI-PAH	Triaromatic steroids	LOD: retene: 25 fg on column	[62]
APLI	Coastal and harbor water	HR TOF, RXI-PAH	48 PAHs (alkylated PAHs in suspected target analysis)	Recovery rate: 60.7% to 157.0%, mean 92.1%	[63]
APLI	Reference materials (urban dust, organics in marine sediment, fresh water harbor sediment, and contaminated soil from a former gas plant site) and environment samples (bituminous coal, suspended particulate matter from river and pine needles)	HR TOF, RXI-PAH	59 PAHs	Recovery: 34%~102%, median, 80% mean 78% LODs: 5~50 fg/μL	[64]
ESI	Human urine	LTQ Orbitrap QQQ Ultra-1	Trimethylsilyl (TMS) derivatives of steroids	LOD 0.5~10 ng/mL	[65]
ESI	Soil	QQQ, DB-EUPAH	PAHs	LOD 0.002~10 μg/mL	[66]

LOD: limit of detection. MD(Q)L: method detection (qualification) limit. ID(Q)L: instrument detection (qualification) limit. CV: coefficient of variance.
